# Venous inflammation might be one of the features of VEXAS syndrome and associated thrombosis

**DOI:** 10.1093/rheumatology/kead168

**Published:** 2023-04-26

**Authors:** Hazan Karadeniz, Mahinur Cerit, Aslıhan Avanoğlu Güler, Abdurrahman Tufan, Yogen Kanthi

**Affiliations:** Division of Rheumatology, Department of Internal Medicine, Gazi University Faculty of Medicine, Ankara, Turkey; Department of Radiology, Faculty of Medicine, Gazi University, Ankara, Turkey; Division of Rheumatology, Department of Internal Medicine, Gazi University Faculty of Medicine, Ankara, Turkey; Division of Rheumatology, Department of Internal Medicine, Gazi University Faculty of Medicine, Ankara, Turkey; National Human Genome Research Institute, National Institutes of Health, Inflammatory Diseases, Bethesda, MD, USA; Division of Intramural Research, National Heart, Lung, and Blood Institute, Bethesda, MD, USA

Rheumatology key message

- VEXAS syndrome is venulitis with severe thickening of the walls of large veins.

Dear Editor, VEXAS (vacuoles, E1 enzyme, X-linked, autoinflammatory, somatic) syndrome is an often fatal, X-linked disease characterized by haematological dysplasia overlapping with severe autoinflammatory manifestations. Acquired somatic mutations of the ubiquitin-like modifier activating enzyme (UBA1) gene impairs ubiquitylation, causing activation of the innate immune system and release of pro-inflammatory cytokines, TNF-α, IL-8, IL-6, IFN-γ and IFN-inducible protein-10 (IP-10) that culminate in severe systemic inflammation [1]. VEXAS syndrome patients present with protean clinical manifestations, including fever, chondritis, neutrophilic dermatosis, arthritis, pulmonary infiltrate and systemic vasculitis [1]. Thrombosis, predominantly as unprovoked venous thrombosis, has been reported in up to 56% of VEXAS syndrome patients [2]. Although chronic inflammation, vasculitis, endothelial dysfunction, disrupted anticoagulant mechanisms and prothrombotic antibodies are suggested mechanisms, the exact pathogenesis of thrombosis is yet to be elucidated [2].

VEXAS syndrome has clinical similarities with Behçet’s syndrome (BS), a systemic vasculitis involving arterial and venous vessels of all sizes. Vascular involvement occurs in 40% of BS patients, mainly as deep vein thrombosis (DVT) [3]. A growing body of evidence suggests that venulitis is the primary pathology responsible for various organ manifestations in BS and other inflammatory diseases [3]. Unfortunately, venous inflammation has received much less attention compared with arteries. Inflammation of veins can be demonstrated by histopathologic examination of affected organs or radiologic examination of large veins. Recently, venous wall thickening (VWT) on Doppler ultrasound has been shown to be a highly sensitive and specific (>80%) finding for the diagnosis of BS [3]. Given the clinical and pathogenetic similarities with BS, we thought venulitis might be the underlying cause of thrombosis in VEXAS syndrome. Herein, we report venulitis in two consecutive VEXAS syndrome patients with the demonstration of severe thickening of the walls of large veins.

Demographic and clinical features of our VEXAS syndrome patients are presented in Table 1. Bilateral lower extremity large veins were examined with Doppler ultrasound in the craniocaudal direction for the presence of thrombi, occlusion, venous insufficiency and arterial occlusive disease. VWT was measured using the methodology described by Alibaz-Oner *et al.* [3]. All measurements were performed three times and the arithmetic mean was given as the result. We also assessed the jugular vein. VEXAS syndrome patients had thickened walls on ultrasound in all examined large veins.

**Table 1.** Demographic, clinical, laboratory and ultrasonographic findings of patients

Characteristics	Patient 1	Patient 2
Sex	Male	Male
Age (years)	73	77
Smoking	Former (30 packs/year)	Never
UBA1 variant	M41L	M41V
Duration of symptoms (years)	2	1
Age at diagnosis (years)	72	77
Comorbidities	COPD	CAD
Clinical features	Fever, weight loss, arthritis, neutrophilic dermatitis, periorbital oedema, pericardial effusion, lung infiltrates, pulmonary embolism, left femoral vein thrombosis and left femoral artery occlusion	Fever, weight loss, arthritis, neutrophilic dermatitis, periorbital oedema, right lower extremity superficial thrombophlebitis, MDS
Haemoglobin (g/dl)	8.8	6.8
MCV (fl)	>110	99.3
ESR (mm/h)	117	135
CRP (mg/l)	88	235
Right CFV thickness^a^ (mm)	0.91	1.43
Left CFV thickness (mm)	1.21	1.50
Right IJV thickness^b^ (mm)	0.57	0.46
Left IJV thickness (mm)	0.70	0.65

a Typical CFV thickness for healthy adult and child populations is <0.5 mm (3–5) and ^b^typical IJV thickness is <0.5 mm for adults (5).

CAD: coronary artery disease; COPD: chronic obstructive pulmonary disease; IJV: internal jugular vein; MCV: mean corpuscular volume; MDS: myelodysplastic syndrome.

Previous studies have shown that venous inflammation causes VWT [3]. Varicosity and DVT are two common causes of VWT. BS is the prototypic disease that primarily targets veins, causing severe VWT both in adults and children [3, 4]. It was reported that BS patients (both adults and children) had increased common femoral vein (CFV) thicknesses [BS *vs* healthy controls, mean 0.77 mm (s.d. 0.34) *vs* 0.27 (0.1)], which is independent of age, disease duration, history of thrombosis and major organ involvement, disease activity, acute phase response and corticosteroid treatment [3]. These measurements were corroborated in other studies [4, 5]. Additionally, VWT in BS seemed significantly greater than non-inflammatory DVT [0.34 mm (s.d. 0.1)] but not significant with APS [0.49 mm (s.d. 0.1)] [3]. Our VEXAS syndrome patients had VWT greater than in BS and APS, suggesting remarkable vein inflammation. Patients with extensive thrombosis (patient 1) had the most severe VWT of the CFV on the affected side, while the latter case (patient 2) had equivalent VWT in both CFVs (Supplementary Fig. S1, available at *Rheumatology* online).

The pathogenesis of organ inflammation in VEXAS syndrome is not entirely understood. UBA1 is essential for the initiation of ubiquitylation, a widely used post-translational protein modification mechanism that regulates many biological processes, including intracellular signalling, proteasomal/lysosomal protein degradation and antimicrobial defence [1]. UBA1 mutations predominantly affect myeloid lineage cells in peripheral blood, which is associated with the activation of multiple inflammatory cytokine pathways and enhanced spontaneous neutrophil extracellular trap formation, all of which are also blamed in the pathogenesis of BS and other thrombotic inflammatory diseases [2]. Consequently, our findings suggest that venulitis might be one of the features of VEXAS syndrome and the underlying mechanism for thrombosis. Further studies are needed to clarify the role of inflammation in venous thrombotic diseases.

## Supplementary Material

kead168_Supplementary_Data

## Data Availability

Data are available upon reasonable request by any qualified researchers who engage in rigorous, independent scientific research, and will be provided following review and approval of a research proposal and Statistical Analysis Plan (SAP) and execution of a Data Sharing Agreement (DSA). All data relevant to the study are included in the article.
