# Supplementary material for: Venous inflammation might be one of the features of VEXAS syndrome and associated thrombosis
Source: Rheumatology (Oxford). 2023 Apr 26;62(9):e269–70. doi: 10.1093/rheumatology/kead168 (PMC10473210; doi:10.1093/rheumatology/kead168)

**Figure S1:** B-mode USG image of the common femoral vein ,juguler vein and vena safena magna. **A.** A healthy individual’s femoral vein ultrasonography, **B.**Vexas patient’s femoral vein (case #1), **C**.Vexas patient’s femoral vein (case# 2).


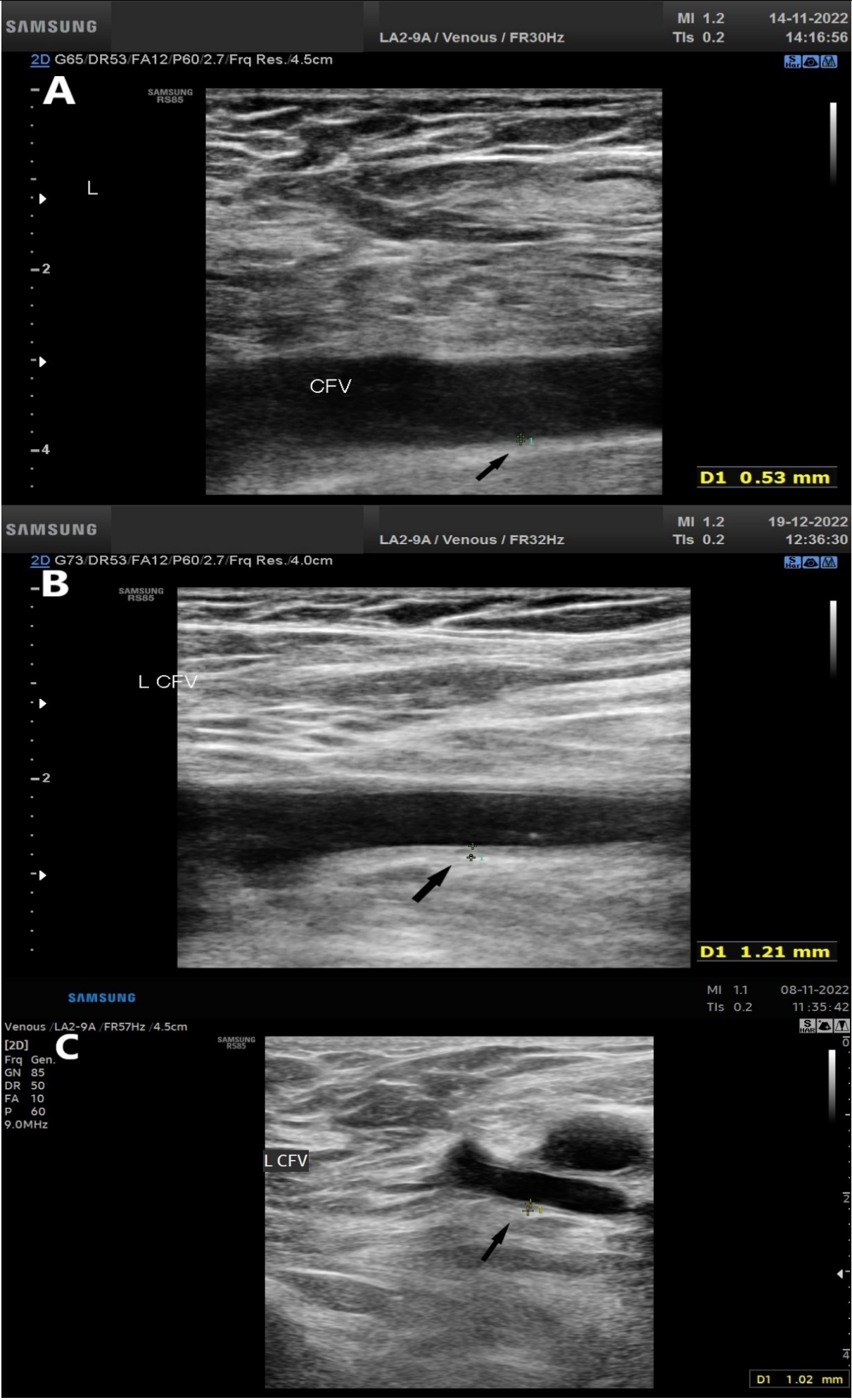

Supplement: kead168_Supplementary_Data [file kead168_supplementary_data.docx]
